# The PPAR*δ* Ligand GW501516 Reduces Growth but Not Apoptosis in Mouse Inner Medullary Collecting Duct Cells

**DOI:** 10.1155/2009/706283

**Published:** 2009-03-04

**Authors:** Jordan Clark, Rania Nasrallah, Richard L. Hébert

**Affiliations:** Department of Cellular and Molecular Medicine, Kidney Research Centre, Faculty of Medicine, University of Ottawa, Ottawa, ON, Canada K1H 8M5

## Abstract

The collecting duct (CD) expresses considerable amounts of PPAR*δ*. While its role is unknown in the CD, in other renal cells it has been shown to regulate both growth and apoptosis. We thus hypothesized that PPAR*δ* reduces apoptotic responses and stimulates cell growth in the mouse CD, and examined the effect of GW501516, a synthetic PPAR*δ* ligand, on these responses in mouse IMCD-K2 cells. High doses of GW501516 decreased both DNA and protein synthesis in these cells by 80%, but had no overall effect on cell viability. Although anisomycin treatment resulted in an increase of caspase-3 levels of about 2.59-fold of control, GW501516 did not affect anisomycin-induced changes in active caspase-3 levels. These results show that a PPAR*δ* ligand inhibits growth but does not affect anisomycin-apoptosis in a mouse IMCD cell line. This could have therapeutic implications for renal diseases associated with increased CD growth responses.

## 1. Introduction

Traditional prostaglandin (PG) signaling in renal epithelial cells occurs through activation of prostanoid receptors [1–3]. In recent years, however, a novel signaling pathway for PGs has been found in which ligand-activated transcription factors called peroxisome proliferator-activated receptors (PPARs) [4] are activated at the nuclear membrane. One PPAR type, PPAR*δ*, also known as PPAR*β*, is ubiquitously expressed in almost every tissue examined [5]. Both prostanoids PGI_2_ and PGE_2_ have been known to activate PPAR*δ* [6, 7]. The main role of PPAR*δ* is in cell survival and this has been well characterized in colon cancer [8–10].

In the kidney, PPAR*δ* is found in every part of the nephron with high levels in the glomerulus, cortical collecting duct, and inner medullary collecting duct (IMCD) and low expression in the outer medullary collecting duct. This PPAR has been implicated in the renal complications of metabolic syndrome [11], but most studies describe a link between PPAR*δ* and survival of renal cells. In a study by Hao et al. [6], PPAR*δ* was shown to increase renal medullary interstitial cell survival during high levels of hypertonic stress, in response to PGI_2_. PPAR*δ* activation also protected kidneys from renal failure after renal ischemia/reperfusion experiments via the antiapoptotic Akt pathway [12]. Although, most of the studies have suggested an antiapoptotic role of PPAR*δ*, a few papers have shown that PPAR*δ* can increase apoptosis [13]. However, PPAR*δ* is recognized as an important protein in the survival of many renal cell types.

PPAR*δ* has also been shown to promote growth responses in a variety of cells. PGE_2_, working through PPAR*δ*, can stimulate proliferation of stem cells [14] and overexpression of PPAR*δ* has been shown to reverse inhibitory growth signals in a prostate epithelial cell line [15]. Furthermore, upregulation of PPAR*δ* in smooth muscle cells promotes cell cycle progression [16]. The effect of PPAR*δ* activation on cell growth in the kidney, however, is unknown.

The collecting duct (CD), specifically the IMCD, survives in a harsh environment due to the hypertonic conditions in the interstitium needed to concentrate urine [17, 18]. In most cells, apoptosis would occur at the levels of stress at which they reside, but the CD manages to survive and resist any apoptotic activity [19]. It is known that cells react to high levels of tonicity by increasing the transport of several osmolytes into the cells aiding in their survival [20]. However, in cells where there is chronic hypertonicity, like the IMCD, the accumulation of these osmolytes may not be sufficient for their continued survival [21]. Cultured IMCD cells have been known to increase levels of cyclooxygenase-2 (COX-2) and stimulate PGE_2_ release up to 33-fold of control in response to hypertonic stress [22]. It is likely then that PGs may contribute to the inherent CD resistance to stress by targeting PPAR*δ* to promote cell survival.

It would appear in most cell lines that PPAR*δ* has opposing growth and apoptotic effects; however, its role in the CD has not been determined. Our study examines the role of PPAR*δ* activation in the survival and growth of IMCD-K2 cells. The mouse IMCD-K2 cell line is derived from the initial portion of the IMCD from a mouse that is transgenic for Simian Virus 40 (SV40) and was shown to retain many characteristics of the intact IMCD, including amiloride-sensitive sodium absorption stimulated by aldosterone [23]. Therefore, we propose that PPAR*δ* activation will regulate growth and apoptotic responses in the mouse IMCD-K2 cells.

## 2. Materials and Methods

### 2.1. IMCD-K2 Cell Culture

Cells were obtained from Dr. Bruce Stanton from Dartmouth Medical College (Hanover, NH, USA). The cells were maintained at 37°C and 5% CO_2_ in DMEM/F-12 supplemented with 10% fetal bovine serum, (FBS, Gibco, Carlsbad, Calif, USA), 1% penicillin/streptomycin (Gibco), ITS (5 *μ*g/mL insulin, 5 *μ*g/mL transferrin, 5 ng/mL selenium; Sigma, St. Louis, Mo, USA), 2 mM L-glutamine (Sigma), and 5 *μ*M dexamethasone (Sigma). Cell culture medium was replaced every 48 hours. Twenty-four hours prior to experiments, the culture medium was replaced with serum-free medium (DMEM/F-12).

### 2.2. Reverse Transcriptase-PCR

RNA was isolated from confluent 100 mm plates of IMCD-K2 cells using 1 mL of the TRIzol reagent (Gibco) according to the manufacturer's instructions and then treated with DNase I (Invitrogen, Carlsbad, Calif, USA). The RNA was reverse transcribed using MuLV reverse transcriptase and random hexamers from the GeneAmp RNA PCR Core Kit (Applied Biosystems, Foster City, Calif, USA) as per the manufacturer's instructions. Next, the RNA was amplified by polymerase chain reaction (PCR), using AmpliTaq DNA polymerase and specific primers for the prostanoid receptors (i.e., EP_1–4_ and IP) and PPAR*δ*. The primers were obtained from the University of Ottawa Biotechnology Research Institute (Ottawa, ON, Canada). The PCR parameters used were 40 cycles of: 95°C for 30 seconds, 59°C for 45 seconds, and 72°C for 60 seconds, followed by 600 seconds at 72°C. The amplification was carried out using the GeneAMP PCR System 2400 (Applied Biosystems). The PCR products were run on an agarose gel and the bands were visualized using ethidium bromide under UV light.

### 2.3. ^3^H-cAMP Assay

Since prostanoids can alter cAMP, their effect on cAMP production in IMCD-K2 cells was determined. The cells were serum starved for 24 hours and stimulated with control (serum-free media), 10^−5^ M forskolin (Sigma) as a positive control, 10^−6^ M cicaprost and iloprost (prostacyclin analogs, Schering Ag Berlin, Berlin, Germany), or 10^−9^–10^−6^ M of prostaglandin E_2_ (PGE_2_, Cayman, Ann Arbor, Mich, USA) The cells were pretreated for fifteen minutes with 0.5 mM isobutylmethylxanthine (IBMX, Sigma), a cAMP phosphodiesterase inhibitor, and 10^−5^ M indomethacin (Sigma), a cyclooxygenase (COX) inhibitor. The stimulation was then stopped with cold 10% trichloroacetic acid for 30 minutes on ice. Four ether extractions were carried out, and a cAMP competitive binding assay was done using the ^3^H-cAMP DPC radioassay (Intermedico, Markham, ON, Canada) as per manufacturer's instructions. The amount of ^3^H-cAMP was measured using a scintillation counter (Beckman Coulter, Mississauga, ON, Canada) and the percent stimulation of cAMP levels over control levels of each sample was calculated.

### 2.4. Western Blotting

IMCD-K2 lysates were obtained by sonicating samples in RIPA lysis buffer containing 1% Nonidet P-40, 1% sodium deoxycholate, 0.1% sodium dodecyl sulphate (SDS, w/v), 4.5 mM NaCl, 2.5 mM Tris (pH 7.4), 8 *μ*M EDTA, 0.2 mM sodium phosphate (pH 7.2), and fresh 0.5 mM PMSF, 1 : 100 protease inhibitor cocktail (Sigma), 1 mM sodium pyrophosphate, 10 mM sodium fluoride, and 100 *μ*M sodium orthovanadate. The protein was quantified using the Bradford reagent method (Bio-Rad, Hercules, Calif, USA). The lysates were then resolved by SDS-PAGE on a polyacrylamide gel using a Mini-PROTEAN II apparatus (Bio-Rad) and transferred onto nitrocellulose membranes (Amersham, Amersham, UK). After blocking for 90 minutes in 10% milk/TBS-T (137 mM NaCl, 20 mM Tris base, 0.1% Tween20), the membranes were incubated overnight with the corresponding primary antibody. The membranes were then incubated with their respective secondary antibodies (i.e., anti-rabbit, anti-mouse, and anti-donkey) for 90 minutes and then washed for 60–90 minutes in TBS-T. The bands were then visualized by ECL. Detection of *β*-actin was used to normalize samples and densitometric analysis of the bands was performed using the Kodak 1D Image Analysis software (Eastman Kodak Company, Rochester, NY, USA).

### 2.5. Real-Time PCR

To determine the effect of PGE_2_ on PPAR*δ* levels in IMCD-K2 cells, RNA was isolated and DNase-treated as described above. The cells were treated for 2, 8, or 24 hours with serum-free media as control, 10^−5^ M indomethacin, or 10^−6^ M PGE_2_. A fifteen-minute pretreatment with indomethacin was performed prior to stimulations with PGE_2_ and forskolin. The mRNA levels of PPAR*δ* were ascertained by real-time PCR using TaqMan One-Step RT-PCR master mix reagents (Applied Biosystems) and an ABI Prism 7000 sequence detection system. Reactions were carried out by using 50 ng of total IMCD-K2 RNA under the following conditions: 48°C for 30 minutes, and 95°C for 10 minutes, and 40 cycles of 95°C for 15 seconds and 60°C for 1 minute. The probe and primers used for mouse PPAR*δ* were forward primer, 5′-GAGCCCAAGTTCGAGTTTGC-3′, reverse primer, 5′-TGAAGAGCGCCAGGTCACT-3′, and probe, FAM-AGTTCAATGCGCTGGAGCTCGATGA-TAM (Sigma Genosys, Oakville, ON, Canada). All values were normalized to GAPDH mRNA levels in the same sample, which was determined by the TaqMan Rodent GAPDH control reagent kit (AppliedBiosystems).

### 2.6. ^3^H-Thymidine Incorporation

To study the effect of the PPAR activation on cell proliferation, DNA synthesis was measured using the incorporation of ^3^H-thymidine. IMCD-K2 cells were cultured in 24-well plates, grown to ∼50% confluence, and then starved with serum-free media. Afterwards the cells were treated for 24 hours with vehicle-treated control (DMSO), or GW501516 (10^−8^, 10^−7^, 10^−6^, 10^−5^ M). GW501516 is among the more specific and most commonly used synthetic PPAR*δ* ligands [24]. ^3^H-thymidine (Amersham, 0.5 *μ*Ci/mL) was added during the final four hours of stimulation. The plates were then washed four times in ice-cold PBS. Next, the cells were permeabilized in 500 *μ*L of 1N NaOH at 37°C for ∼30 minutes, and the amount of ^3^H-thymidine in counts per minute (cpm) was measured using a scintillation counter. Samples were done in triplicate and thymidine incorporation is expressed as fold control.

### 2.7. ^3^H-Leucine Incorporation

To study the effect of the PPAR ligands on cell growth, protein synthesis was measured using the incorporation of ^3^H-leucine. IMCD-K2 cells were cultured in 24-well plates, grown to ∼50% confluence, and then starved with serum-free media for 24 hours. Next the cells were stimulated for 24 hours with vehicle-treated control (DMSO) and GW501516 (10^−8^, 10^−7^, 10^−6^, and 10^−5^ M). ^3^H-leucine (Amersham, Perkin Elmer, Waltham, Mass, USA, 0.5 *μ*Ci/mL) was added to each well during the 24-hour stimulation. The cells were then prepared as described above and the amount of ^3^H-leucine in counts per minute was measured using a scintillation counter. Samples were done in triplicate and leucine incorporation is expressed as fold control.

### 2.8. Cell Viability

To evaluate the effect of GW501516 on the viability of IMCD-K2 cells in both the presence and absence of a death response, a cell viability assay was performed. IMCD-K2 cells were grown to 70% confluence in 96-well plates and stimulated for 18 hours with control (1 *μ*L of ethanol and 1 *μ*L DMSO in DMEM/F-12), GW501516 (10^−8^, 10^−7^, 5 × 10^−7^, 10^−6^, 5 × 10^−6^, and 10^−5^ M), anisomycin (Sigma; 100, 1000 ng/mL), or cisplatin (Sigma; 50 ng/mL). Some cells were treated with both anisomycin and GW501516 (10^−6^ M) following a 24-hour pretreatment with GW501516 (10^−6^ M). Anisomycin and cisplatin were used to induce cell death. Following the stimulation, the cells were incubated in 100 *μ*L of a 1x DNA dye-binding solution from the CyQAUNT NF kit (Invitrogen). The fluorescence intensity, representing the number of cells, was measured in a FLUOstar Galaxy plate reader (BMG Labtechnologies, Durham, NC, USA). Samples were done in triplicate and fluorescence intensity is expressed as fold control.

### 2.9. Colorimetric Caspase-3 Activity Assay

To determine if GW501516 is involved in apoptosis, a caspase-3 activity assay was carried out using the colorimetric CaspACE Assay System kit (Promega, Madison, Wis, USA), as per manufacturer's instructions. IMCD-K2 cells were grown to ∼50% confluence in 100 mm plates and starved for 24 hours. Next the cells were treated with control (1 *μ*L of ethanol and 1 *μ*L of DMSO in 1 mL of DMEM/F-12), 10% FBS, GW501516 (10^−6^ M), anisomycin (250 ng/mL), or Z-VAD-FMK (2 × 10^−5^ M), a pan-caspase inhibitor supplied in the kit. Some cells were treated with both anisomycin and GW501516 following a 24-hour pretreatment with GW501516. Also, some plates were treated with anisomycin and Z-VAD-FMK following a 24-hour pretreatment with the inhibitor. Following stimulations, the cells were resuspended in 100–200 *μ*L of the caspase lysis buffer subjected to two freeze-thaw cycles (at −20°C), and a 15-minute incubation on ice. The lysates were then centrifuged for 20 minutes at 13 100 revolutions per minute and protein was quantified using the Bradford method. 50 *μ*g of each sample was incubated with the colorimetric caspase-3 substrate Ac-DEVD-pNA (2 × 10^−4^ M) in a 96-well plate at room temperature overnight. The endogenous active caspase-3 bound to the substrate was determined by the absorbance at 405 nm (FLUOstar Galaxy). Samples were done in duplicate and the caspase activity is expressed as relative absorbance.

### 2.10. Tunel Assay

To further evaluate the effect of GW501516 on apoptosis, a terminal deoxynucleotidyl transferase biotin-dUTP nick end labeling (TUNEL) assay was performed. IMCD-K2 cells were grown to ∼60% confluency on glass coverslips in a 24-well plate and treated in duplicate for 24 hours with control (1 *μ*L of ethanol and 1 *μ*L of DMSO in 1 mL of DMEM/F-12), GW501516 (10^−8^, 10^−6^ M), or anisomycin (250 ng/mL). Some cells were treated with both GW501516 (10^−8^, 10^−6^ M) and anisomycin, following a 3-hour pretreatment with the respective concentrations of GW501516. After the stimulation, the cells was fixed in 4% paraformaldehyde and 0.2% picric acid in 0.16 M sodium phosphate buffer (pH 6.9). The coverslips were then washed with PBS three times and incubated with 2 *μ*L CoCl_2_, 5 *μ*L 5X TDT buffer, 0.1 *μ*L terminal transferase (all taken from the Roche Terminal Transferase recombinant kit), 0.17 *μ*L biotin-16-dUTP (Roche, Mississauga, ON, Canada), and 17.73 *μ*L of H_2_O for 1 hour at 37°C in a humid chamber. Next, the cells were washed with 4X SSC (0.6 M NaCl, 76 mM Na Citrate, pH 7), blocked for 30 minutes with 1% milk/4X SSC at room temperature, and incubated with streptavidin-Cy2 (Molecular Probes,Carlsbad, Calif, USA, 1.0 mg/mL) at 1 : 1000 for 45 minutes at room temperature in the dark. The coverslips were then washed with PBS and incubated with DAPI for 45 minutes at 1 : 1000. Following three more washes, the coverslips were inverted onto Fluoromount mounting media (Electron Microscopy Sciences, Hatfield, Pa, USA) on glass slides. The cells were visualized with the fluorescent microscope Axioskop Mot 2 (Zeiss, Oberkochen, Germany) and captured with the camera AxioCam (Zeiss). For each coverslip, 8 fields of view at 200x magnification were examined using pictures from both the DAPI filter (excitation: ∼360 nm and emission: ∼450 nm) and the green filter (excitation: ∼480 nm and emission: ∼540 nm). The pictures were processed using the Axiovision software (Zeiss). The terminal transferase and biotin-16-dUTP target-free DNA fragments of apoptotic cells produce a fluorescent green signal. The number of apoptotic cells was counted, and from the DAPI filter a percentage of TUNEL-positive cells were determined. A total of 16 fields of view, or two coverslips, were used for each sample.

### 2.11. Statistics

The GraphPad Prism software (La Jolla, Calif, USA) for Windows (version 4.02) was used to analyze data. Results are expressed as means ± standard error of the mean (S.E.M). Either an unpaired *t*-test or a one-sample *t*-test (with a hypothetical value of 1) was used to evaluate the statistical significance between data points. A *P*-value < .05 was considered statistically significant.

## 3. Results

### 3.1. EP Receptor Subtypes and PPAR*δ* Expression

**Figure 1:**
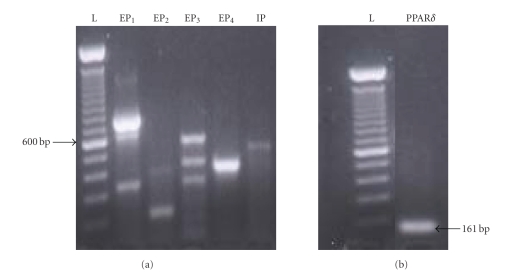
EP receptor subtypes and PPAR*δ* are expressed in IMCD-K2 cells. RNA was isolated from IMCD-K2 cells using TRIzol, reverse transcribed, amplified by polymerase chain reaction using primers for (a) the prostanoid receptors and (b) PPAR*δ* receptor, and visualized on an agarose gel using ethidium bromide under ultraviolet light. As shown, specific bands for each PCR product were obtained at the predicted size for EP_1_ (336 base pair), EP_3_ (437 base pair; plus bands at ∼350 base pair and ∼600 base pair), and EP_4_ (423 base pair), as well as PPAR*δ* (161 base pair).

PGE_2_ is known to activate prostanoid receptors, EP_1_, EP_2_, EP_3_, and EP_4_ as well as PPAR*δ*. Thus, the presence of these receptors in IMCD-K2 cells was determined. PCR was performed and bands corresponding to the prostanoid and PPAR*δ* receptors were visualized as shown in Figures 1(a) and 1(b), respectively. Previous studies in our laboratory demonstrated that at higher stringency the band in the EP_2_ lane disappears and that the product detected with the IP primers, present at approximately 200 base pair (bp) greater than the predicted size of 407 bp, does not correspond to the IP receptor, as determined by cloning and sequencing. The band seen between 700 and 800 bp in the EP_1_ lane represents protein kinase N (PKN), as the genes for PKN and EP_1_ overlap [25]. The multiple bands seen in the EP_3_ are most likely due to alternative splicing of the EP_3_ gene [26]. The products were not present when the reverse transcriptase was omitted (negative control; data not shown). These results indicate that IMCD-K2 cells express EP_1_, EP_3_, EP_4_, and PPAR*δ*.

### 3.2. Effect of PGE_2_ on Camp Stimulation

**Figure 2:**
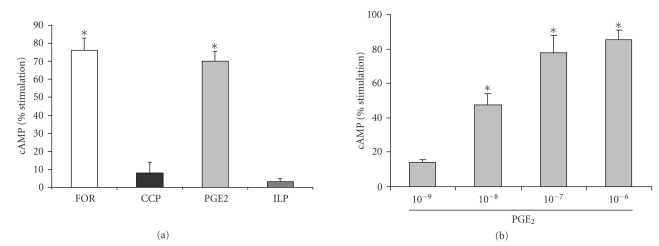
PGE_2_ stimulates cAMP in IMCD-K2 cells.Serum-starved cells were stimulated for ten minutes with (a) 10^−6^ M of different prostanoids (cicaprost (CCP), iloprost (ILP), and PGE_2_) or 10^−5^ M forskolin (FOR), and with (b) differing concentrations of PGE_2_ (10^−9^ to 10^−6^ M). A competitive binding assay with ^3^H-cAMP was performed and the percent cAMP stimulation was determined using a scintillation counter. Values are means ± S.E.M.; *n* = 3–4. *P < .05 compared to control.

Both the EP_3_ and EP_4_ receptors elicit changes in intracellular cAMP; therefore, the effect of different prostanoids on cAMP stimulation was examined. Forskolin was used as a positive control. As shown in Figure 2(a), prostacyclin (PGI_2_) analogs cicaprost (CCP) and iloprost (ILP) did not alter cAMP levels. PGE_2_, however, did cause a significant increase in percent stimulation, at about 70% above control, comparable to forskolin. As shown in Figure 2(b), treatment with increasing amounts of PGE_2_ resulted in a concentration-dependent increase in cAMP, from 14.0 to 85.5%.

### 3.3. The Effect of PGE_2_ on PPAR*δ* Expression

**Figure 3:**
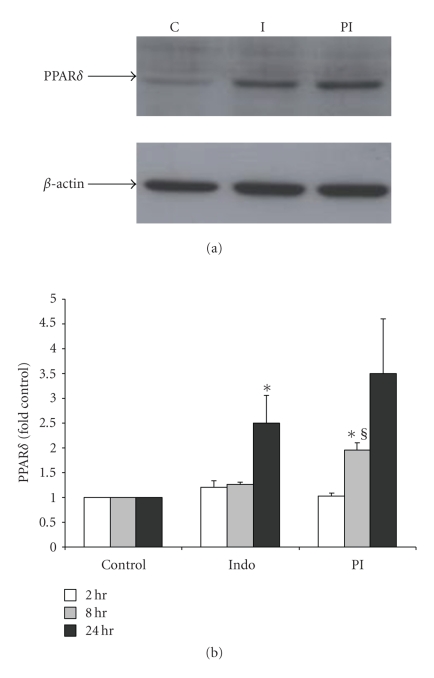
PGE_2_ increases PPAR*δ* protein expression. Protein was isolated from IMCD-K2 cells that had been treated with serum-free media (control, C), 10^−5^ M indomethacin (Indo, I), or 10^−6^ M PGE_2_ in the presence of I (PI) 2, 8, and 24 hours. The proteins were (a) run on an SDS-PAGE gel and Western blotted using an anti-PPAR*δ* antibody (1 : 500). A representative blot of 24-hour treatment is shown. The membranes were stripped and *β*-actin was detected to normalize samples for (b) densitometric analysis. Expression is presented as fold of control. Values are means ± S.E.M.; *n* = 3–6. *P < .05 compared to control; §*P* < .05 compared to indomethacin.

**Figure 4:**
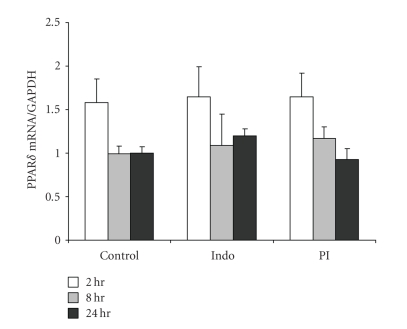
PGE_2_ does not affect PPAR*δ* mRNA expression. RNA was isolated from IMCD-K2 cells using the TRIzol method. The cells had been treated for 2, 8, and 24 hours with serum-free media (control), 10^−5^ M indomethacin (Indo), or 10^−6^ M PGE_2_ in the presence of 10^−5^ M indomethacin (PI). The RNA samples were quantified using real-time PCR using primers and probes for PPAR*δ*. Glyceraldehyde-3-phosphate dehydrogenase (GAPDH) was used as an internal control. Expression is presented as the amount of PPAR*δ* mRNA expression divided by GAPDH expression. Values are means ± S.E.M.; *n* = 5.

Since PGE_2_ has been known to activate PPAR*δ* to regulate several growth processes [7, 14], it was ascertained that if PGE_2_ stimulation could affect PPAR*δ* expression in IMCD-K2 cells. Indomethacin, a COX inhibitor, was used to inhibit endogenous PG synthesis. As shown in Figures 3(a) and 3(b), treatment with PGE_2_, in the presence of indomethacin, for 8 hours resulted in a significant increase, of ∼2-fold, in PPAR*δ* protein expression compared to control or indomethacin alone, as measured by Western Blotting. Stimulation for 24 hours also resulted in increased PPAR*δ* protein expression, but this increase was not significant when compared with indomethacin alone. A representative blot for 24 hours is shown in Figure 3(a). However, two-hour stimulation did not alter the protein levels of PPAR*δ*. We also examined the effect of PGE_2_ on PPAR*δ* mRNA expression by real-time PCR. As shown in Figure 4, PPAR*δ* mRNA remained unchanged with any treatment group or exposure time. Forskolin had no effect on expression either (data not shown) indicating that cAMP does not alter PPAR*δ* protein or RNA expression.

### 3.4. The Effect of GW501516 on Cell Proliferation/Growth

**Figure 5:**
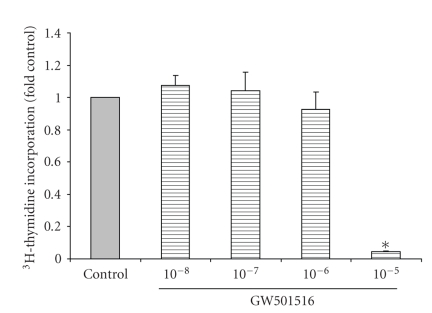
GW501516 causes a decrease in DNA synthesis at high doses. Serum-starved cells were stimulated for 24 hours with serum-free media (control) or increasing concentrations of GW501516 (10^−8^ to 10^−5^ M) ^3^H-thymidine was added to IMCD-K2 cells while they were being stimulated and thymidine incorporation was measured in counts per minute using a scintillation counter, and expressed as fold control. Values are mean ± S.E.M.; *n* = 3. *P < .05 compared to control.

**Figure 6:**
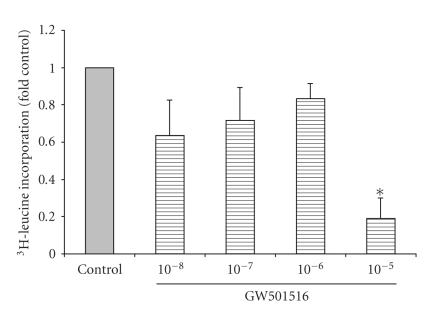
GW501516 causes a decrease in protein synthesis at high doses. Serum-starved cells were stimulated for 24 hours with serum-free media (control) or increasing concentrations of GW501516 (10^−8^ to 10^−5^ M) ^3^H-leucine was added to IMCD-K2 cells while they were being stimulated and leucine incorporation was measured in counts per minute using a scintillation counter, and expressed as fold control. Values are mean ± S.E.M.; *n* = 3–5. *P < .05 compared to control.

PPAR*δ* has been known to affect cell proliferation; therefore, we studied the effect of the PPAR*δ* agonist, GW501515, on the proliferation/growth of IMCD-K2 cells. As shown in Figure 5, 10^−5^ M GW501516 reduced ^3^H-thymidine incorporation to about 0.05-fold control. PPAR*δ* also regulates cell growth and as shown in Figure 6, 10^−5^ M GW501516 produced a significant reduction in ^3^H-leucine incorporation to approximately 0.19 of control. At lower concentrations of GW501516, neither DNA nor protein synthesis was altered.

### 3.5. The Effect of GW501516 on Cell Viability

**Figure 7:**
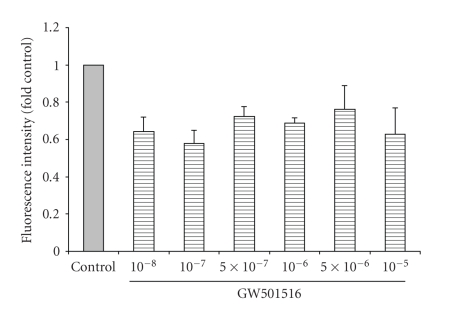
GW501516 does not significantly alter IMCD-K2 cell viability. Serum-starved cells were stimulated for 24 hours with serum-free media (control) or increasing concentrations of GW501516 (10^−8^ to 10^−5^ M). The cells were then incubated for one hour with the DNA dye-binding solution from the CyQUANT NF assay kit (Invitrogen). Fluorescence intensity, representing number of cells, was then measured as per the manufacturer's instructions. Values (fold control) are means ± S.E.M.; *n* = 3.

**Figure 8:**
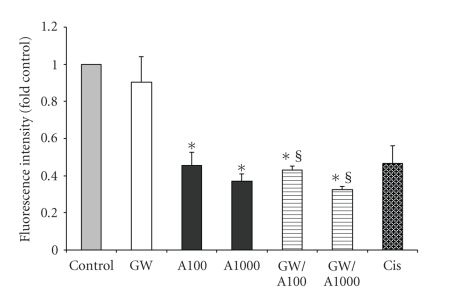
GW501516 has no effect on anisomycin-induced cell death. Serum-starved cells were stimulated with serum-free media (control), 10^−6^ M GW501516 (GW), 100 and 1000 ng/mL anisomycin (A100 and A1000, resp.), or 50 ng/mL cisplatin (Cis) for 18 hours. Some of the cells treated with anisomycin were pretreated with 10^−6^ GW501516 for 24 hours and cotreated with the agonist and anisomycin for 18 hours (GW/A100 and GW/A1000). The cells were then incubated for one hour with the DNA dye-binding solution from the CyQUANT NF assay kit (Invitrogen). Fluorescence intensity, representing number of cells, was then measured as per the manufacturer's instructions. Values are means ± S.E.M.; *n* = 3. *P < .05 compared to control; §*P* < .05 compared to GW.

To further characterize the effect of GW501516 on IMCD-K2 cells and determine if it has a role in cell survival, a cell viability assay was carried out. As shown in Figure 7, GW501516 (10^−8^, 10^−7^, 5 × 10^−7^, 10^−6^, 5 × 10^−6^, and 10^−5^ M) caused a decrease in cell viability between 0.8 and 0.6-fold of control but none of the values was significant. On its own, GW501516 did not induce death in the IMCD-K2 cells. To examine if the PPAR*δ* agonist could protect cells from an induced death, IMCD-K2 cells were treated with both anisomycin and GW501516. Cisplatin (*cis*-diamminedichloridoplatinum[II]) was used as an additional positive control for cell death. As shown in Figure 8, anisomycin (100 and 1000 ng/mL) caused cell viability to decrease to approximately 0.46- and 0.37-fold of control, respectively. However, 10^−6^ M GW501516 did not significantly alter the decrease in cell viability seen with the addition of either 100 or 1000 ng/mL of anisomycin.

### 3.6. The Effect of GW501516 on Caspase Activity

**Figure 9:**
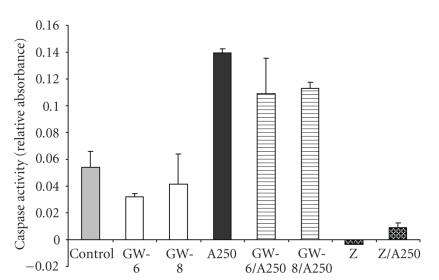
Anisomycin increases caspase activity in IMCD-K2 cells, independent of GW501516. Serum-starved cells were treated with serum-free media (control) for 24 hours, 250 ng/mL anisomycin (A250) for 24 hours, 10^−6^ M GW501516, and 10^−8^ M GW501516 for 24 hours pretreatment followed by 10^−6^ M and 10^−8^ M GW501516 for 24 hours with (GW-6/A250 and GW-8/A250) or without anisomycin (GW-6 and GW-8), Z-VAD-FMK, a pan-caspase inhibitor with (Z/A250) or without (Z) anisomycin. Values are mean ± S.E.M.

Besides having effects in cell growth, PPAR*δ* has also been linked to changes in apoptosis. Therefore, the antiapoptotic effect of GW501516 was evaluated in IMCD-K2 cells. Caspase-3 activity was examined because it is a downstream effector protease that is common to all apoptotic pathways. As shown in Figure 9, anisomycin treatment resulted in an increase of caspase-3 levels of about 2.59-fold of control. Pre/cotreatment with GW501516 showed a decrease that was 0.79-fold (10^−6^ M) and 0.81-fold (10^−8^ M) of anisomycin alone. GW501516 alone showed no considerable change in caspase activity. The caspase inhibitor, Z-VAD completely abolished caspase activity in unstimulated cells, and in anisomycin-treated cells it decreased caspase activity to 0.06-fold of anisomycin alone.

**Figure 10:**
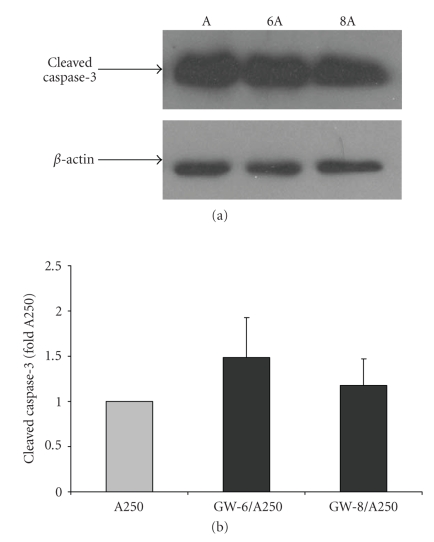
Pretreatment with GW501516 does not significantly alter anisomycin-induced cleaved caspase-3 protein in IMCD-K2 cells. Protein was isolated from IMCD-K2 cells that had been treated with control serum-free media, 10^−6^ or 10^−8^ M GW501516, 250 ng/mL anisomycin (A250 or A) for 24 hours, 10^−6^ M GW501516 for 3 hours pretreatment followed by 10^−6^ M GW501516 for 24 hours with A250 (GW-6/A250), or 10^−8^ M GW501516 for 3 hours pretreatment followed by 10^−8^ M GW501516 for 24 hours with A250 (GW-8/A250). (a) The lysates were run on an SDS-PAGE gel and incubated with an anticleaved caspase-3 antibody (1 : 1000). (b) The membranes were stripped and incubated with anti-*β*-actin antibody (1 : 10000) to normalize for densitometry. Expression is presented as fold A250. Values are mean ± S.E.M.; *n* = 8.

Activation of caspase was also measured by Western Blotting using a cleaved caspase-3 antibody. As shown in Figure 10(a), a 17 kDa band corresponding to cleaved caspase-3 was observed for the lysates that had been treated with anisomycin. The intensity of the bands in the lanes representing control and GW501516 alone was too low to be analyzed in the majority of the experiments; therefore, densitometric analysis was not performed for those samples. The levels of active caspase-3 (see Figure 10(b)) increased to 1.48 and 1.18 of anisomycin alone with both cotreatments (10^−6^ and 10^−8^ M GW501516, resp.) but the difference was not significant.

### 3.7. The Effect of GW501516 on Apoptosis

**Figure 11:**
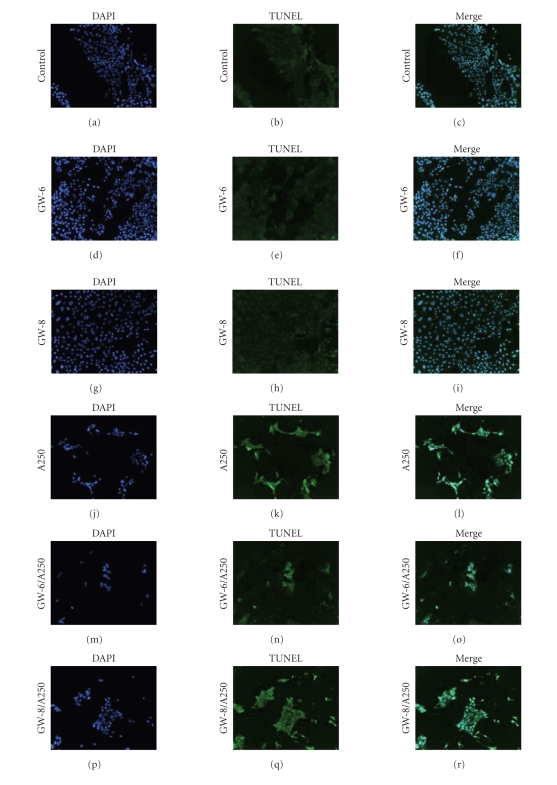
GW501516 pretreatment has no effect on anisomycin-induced apoptosis. Serum-starved cells were stimulated with (a)–(c) serum-free media (control), (d)–(f) 10^−6^ M GW501516 (GW-6), (g)–(i) 10^−8^ M GW501516 (GW-8), (j)–(l) 250 ng/mL anisomycin (A250) for 24 hours, (m)–(o) 10^−6^ M, or (p)–(r) 10^−8^ M GW501516 for 3 hours pretreatment followed by 10^−6^ M or 10^−8^ M GW501516 for 24 hours with A250 (GW-6/A250 and GW-8/A250). The cells were incubated with terminal transferase and biotin-16-dUTP for one hour, followed by two 45-minute incubations with streptavidin-Cy2 and anti-DAPI. The cells were then mounted on slides, visualized with a fluorescent microscope, and captured with a camera. Apoptotic cells are shown in green, while the blue represents DAPI-positive cells.

In addition to looking at the effect of GW501516 on caspase activity, TUNEL assays were performed to further evaluate the apoptotic response. As shown in Figure 11, the proportion of TUNEL-positive cells increased while the total number of cells decreases with the addition of 250 ng/mL anisomycin when compared to control or the PPAR*δ* agonist (at both concentrations). Pre/cotreatment of 10^−6^ M or 10^−8^ M GW501516 with anisomycin was comparable to anisomycin alone.

## 4. Discussion

### 4.1. PGE_2_ and PPAR*δ*

It has been shown previously that prostaglandins, including PGE_2_, and PPARs influence one another's activity [27–30]. Although our studies showed that the PGE_2_ receptors EP_1_, EP_3_, and EP_4_ are present in the IMCD-K2 cells and PGE_2_ produces an increase in cAMP production, consistent with the expression of EP_4_, PGE_2_ had no effect on PPAR*δ* mRNA expression. However, treatment with PGE_2_ for eight hours did elicit an increase in PPAR*δ* protein levels. There are two likely explanations for this. The first reason is that the PPAR*δ* protein could be stabilized. A previous study in a prostate cancer cell line has shown that PGE_2_ can stabilize hypoxia-inducible factor 1 alpha (HIF-1*α*) protein levels, without affecting mRNA levels [31]. This would most likely be due to PGE_2_ preventing proteolysis of PPAR*δ*. Interestingly, it has been revealed that unlike most nuclear receptors, degradation of PPAR*δ* does not occur upon ligand binding but is in fact inhibited by it [32]. This would indicate that PGE_2_ could be causing an increase in PPAR*δ* activation. The second possible role of PGE_2_ is that it could be enhancing the translation of basal PPAR*δ* mRNA levels or stabilizing it. The ability of PGE_2_ to stabilize mRNA (interleukin 8) has been previously described in a paper by Yu and Chadee [33]. Therefore, from the results reported here and the literature, the effect of PGE_2_ on PPAR*δ* is most likely a posttranscriptional event. We also observed an increase in PPAR*δ* protein in response to 24 hours indomethacin. It is not clear at this time whether this effect is due to a direct increase in expression, but it has previously been shown that PPAR expression is altered by NSAIDs [34].

### 4.2. PPAR*δ* Agonist and Cell Proliferation/Growth in IMCDK2 Cells

It has been previously reported that activation of PPAR*δ* and the use of PPAR*δ* ligands promote cell survival and proliferation. One recent study, using both PPAR*δ* short interfering RNA (siRNA) and a PPAR antagonist, showed that downregulation or blocking the activation of PPAR*δ*, respectively, inhibited the PGE_2_-induced proliferation of mouse embryonic stem cells [14]. Another study revealed that a PPAR*δ* agonist can stimulate propagation in both human and mouse aortic endothelial cells [35]. However, the effects of PPAR*δ* on renal cell growth responses have not been characterized. Here we show that a highly specific PPAR*δ* ligand, GW501516, decreased DNA and protein synthesis in IMCD-K2 cells, but only at high concentrations. Although most studies indicate that PPAR*δ* activation results in stimulation of cell growth, mostly in human and mouse colon cancer cells [7, 36], there have been previous findings showing that PPAR*δ* agonists, including GW501516, can hinder or have no effect on cell growth in certain human breast cancer cells and melanomas [37, 38]. These differences may be due to species differences or diversity among cell lines.

### 4.3. PPAR*δ* Agonist and Anisomycin-Induced Apoptosis in IMCD-K2 Cells

Another finding in our study is that treatment with GW501516 does not reverse the reduction in cell survival due to exposure to anisomycin. These results are inconsistent with the literature that demonstrates that activation of PPAR*δ*, or the use of a PPAR*δ* agonist, protects many different cell types, including renal cells [12]. It is possible that the agonist may not actually be activating PPAR*δ* in IMCD-K2 cells, as its efficacy in this cell line has never been tested. In addition, PPAR*δ* activation may not be affecting cell survival if the death response is mostly through necrosis because studies only show a survival role for PPAR*δ* with regard to apoptosis. To evaluate if the agonist is having an effect on PPAR*δ* activation, experiments can be performed with cells transfected with a PPRE-driven reporter plasmid [39]. To completely assess death pathways activated by anisomycin, flow cytometry using the annexin V-FITC and propidium iodide stains should be used [40].

Even though the use of the agonist, in our experiments, elicits no response in overall cell survival in IMCD-K2 cells, the effect on apoptosis had yet to be investigated. When the CD is in a disease state due to urinary tract obstruction, ischemia-reperfusion injury or other insults apoptosis levels tend to increase [41, 42]. Thus, we wished to examine if PPAR*δ* could protect the CD from apoptosis. Using a variety of experiments assessing apoptosis (caspase activity assay, Western blotting, TUNEL), we found no significant change in the levels of apoptosis in cells treated with GW501516 compared to those stimulated with just anisomycin. The variation between our data and the majority of the literature could be due to differences in cell types and the condition of the cells, as most studies have focussed on colorectal cancer tissues [7, 8, 43, 44]. It seems unlikely that the discrepancy in PPAR*δ* effects is due to species differences as most of the cancer studies were done using mouse tissues. However, a few studies clearly indicate that PPAR*δ* may not provide any prevention in certain types of apoptosis, such as in the López et al. [45] study where overexpression of PPAR*δ* did not protect against aspirin-induced apoptosis in Jurkat cells. Thus, it is possible that PPAR*δ* may only protect cells from apoptosis in certain conditions and cell types. From the data we collected, GW501516 has no effect on cell survival or anisomycin-induced apoptosis. The IMCD and possibly the IMCD-K2 cells are resistant to cell death because their environment in vivo is one of hypertonicity [46]. This inherent resiliency may make them resistant to different treatments, similar to that previously observed in a mouse IMCD cell line [47].

### 4.4. Summary

The main objective of this study was to determine whether PPAR*δ* regulates growth and apoptosis in IMCD-K2 cells. PPAR*δ* is highly expressed in the CD, and thus may be responsible for the protection of the CD. We showed that growth responses, including DNA and protein synthesis, in these cells are reduced by PPAR*δ* ligands, but only at high doses. GW501516 had no effect on anisomycin-induced apoptosis in the IMCD-K2 cell line.
